# VOICES OF CARE: INSIGHTS FROM DIVERSE CAREGIVERS

**DOI:** 10.1093/geroni/igae098.1101

**Published:** 2024-12-31

**Authors:** Barbara Mendez Campos, Karen Lyons

**Affiliations:** Chair; Discussant

## Abstract

Recognized as the backbone of the U.S. healthcare system, informal caregivers face unprecedented challenges amidst an ongoing care crisis. Understanding the profound effects of informal care responsibilities is critical to the health and well-being of our society. According to AARP, in 2020, the number of individuals engaged in caregiving for an ill or disabled family member surpassed 53 million, up nearly 10 million since 2015. There is increasing evidence documenting that social inequalities often compound the negative health and financial outcomes for caregivers as they age, making research that focuses attention on the racial, ethnic, and cultural contexts that shape not only the caregiving experience, but also the outcomes that result, central to our ability to develop interventions. This symposium delves into the intricate realities of caregiving, providing valuable insights into economic, cultural, and health-related dimensions. It proposes solutions to positively influence the experiences of older adults, their families, and the broader healthcare system. The first paper explores the enabling factors and barriers African American women caregivers confront in seeking support. The second paper evaluates a digital storytelling intervention’s impact among African American caregivers. The third paper investigates the repercussions of caregiving on older workers’financial, health, and employment outcomes during the COVID-19 pandemic. The final paper reports a scoping review on the role of interpersonal factors in shaping health behaviors among Hispanic older adults. A discussant will reflect on these studies, underscoring the imperative for ongoing research encompassing diverse voices, methodologies, and the impacts on informal caregivers. Family Caregiving Interest Group Sponsored Symposium

